# A multi-centre study on epidemiology and clinical characteristics of human metapneumovirus infection in Sri Lanka from 2021 to 2024

**DOI:** 10.1099/acmi.0.001022.v3

**Published:** 2025-08-29

**Authors:** Shiyamalee Arunasalam, Ishani De Silva, Udeshika Sathgurupathi, Veranja Liyanapathirana, Wasana Kudagammana, Faseeha Noordeen, Thulani Pattiyakumbura, Saranga Sumathipala, Rohitha Muthugala

**Affiliations:** 1 Department of Microbiology, Faculty of Medicine, University of Peradeniya, Peradeniya 20400, Sri Lanka; 2 Teaching Hospital Peradeniya, Peradeniya 20400, Sri Lanka; 3 Public Health Laboratory, Vavuniya, 43000, Sri Lanka; 4 Regional Virology Laboratory, Teaching Hospital Anuradhapura, Anuradhapura, 50000, Sri Lanka; 5 Regional Virology Laboratory, National Hospital Kandy, Kandy, 20000, Sri Lanka; 6 Genomic Laboratory, National Cancer Institute Sri Lanka, Maharagama, 10230, Sri Lanka; 7 Medical Research Institute, Colombo, 00800, Sri Lanka

**Keywords:** acute respiratory tract infection, epidemiology, human metapneumovirus, Sri Lanka

## Abstract

**Introduction.** Human metapneumovirus (hMPV), first identified in 2001, is one of the major respiratory pathogens causing acute respiratory tract infections (ARTIs). In Sri Lanka, data on epidemiology and clinical characteristics of hMPV infections are limited. In this study, we aimed to investigate the epidemiology and clinical characteristics of hMPV infection in adults and children with ARTIs in different locations in Sri Lanka from January 2021 to December 2023.

**Methods.** A total of 1,582 respiratory samples from patients with ARTIs were enrolled from four tertiary care hospitals. Nasopharyngeal swab samples were subjected to real-time reverse transcriptase PCR to test for hMPV using a commercial multiplex assay. Demographic and clinical data were extracted from the patients’ clinical records. A selected subset of positive samples was subjected to genomic sequencing using an amplicon-based approach with the Respiratory Pathogen ID/AMR Library Prep and Enrichment Kit using the Illumina platform.

**Results.** hMPV infection was identified in 1.64% (26/1,582) of patients, with the majority being children under 5 years of age. The co-infection rate was 0.34% with other respiratory viruses. The most common clinical presentation in hMPV infection included acute upper respiratory tract infection with fever, cough and cold and sore throat.

**Conclusion.** hMPV is an important respiratory pathogen in children, causing ARTIs. hMPV-infected patients showed a range of respiratory symptoms with varying severity ranging from common cold to life-threatening lower respiratory tract infections. Continuous surveillance on hMPV infection may help in monitoring the hMPV activity, which will help in tracking the emergence of hMPV infections.

## Data Summary

The authors confirm that all supporting data have been provided within the article. Genomic sequencing data are available in the National Center for Biotechnology Information (NCBI) database (GeneBank accession no. PV178132). A supplementary file summarizing demographic and clinical details of the hMPV positive samples is also provided along with this article.

## Introduction

Human metapneumovirus (hMPV) is a respiratory virus that has been known to cause acute respiratory tract infections (ARTI) since its identification in 2001 [1]. hMPV has gained increased attention after the recent surge in hMPV-associated ARTI cases in different countries, including China, United Kingdom (UK), India and Malaysia. China has reported a rise in hMPV cases, particularly among individuals under the age of 14 by second of January 2025 [2]. In the UK, as of 19 January 2025, hMPV was detected in 4.9% of hospital patients tested for respiratory infections, which is slightly higher than the previous year’s peak of 4.18% [3]. In India, seven cases have been confirmed across multiple states, including Karnataka, Gujarat and Tamil Nadu, following the hMPV outbreak reported in China in early 2025 [4]. Malaysia also recorded 327 hMPV cases in 2024, which represents a 45% increase compared to 225 cases in 2023 [5]. However, health officials monitoring the respiratory illnesses at global, regional and country levels through collaborative surveillance systems have not made emergency declarations yet [6].

hMPV is a non-enveloped negative-sense RNA virus of the family *Pneumoviridae* and genus *Metapneumovirus*. hMPV is likely to have originated from animals infecting only humans [7]. There are two major types of hMPV, identified as hMPV-A and -B. These are further divided into four subtypes, named as A1, A2, B1 and B2, and two additional subtypes of A2 named as A2a and A2b [1]. hMPV reaches its optimal infectivity within 4–6 days of infection [8]. hMPV is transmitted through direct contact with contaminated body secretions like saliva, aerosol and droplets and through contaminated surfaces. Moreover, there have been reports of nosocomial hMPV infections in infants [9].

hMPV typically causes mild respiratory symptoms similar to the common cold or flu, such as cough, fever and nasal congestion. However, it can cause more severe illness in young children, the elderly and immunocompromised individuals [10]. Experts recommend practising good hygiene, such as frequent handwashing, covering coughs and sneezes and avoiding close contact with sick individuals, to reduce its spread [11]. There is no specific antiviral treatment or vaccine against hMPV infection, but most cases resolve on their own with supportive care [12].

Despite its known prevalence worldwide, there is emerging data on hMPV from different areas of Sri Lanka. Since the first report of hMPV infection in Sri Lanka in 2013 from a 9-month-old girl presented to the outpatient department of a teaching hospital, a few studies have focused on the correlation of hMPV with ARTI, including a mini hMPV wave with severe acute respiratory tract infection (SARI) [13,17]. The present study aims to fill this gap by investigating the prevalence and clinical characteristics of hMPV infection from January 2021 to December 2023 in different locations in Sri Lanka, aiming to enhance public health strategies and preparedness for respiratory infection outbreaks.

## Methods

### Study design and setting

The study was conducted as a prospective descriptive study in a sample of patients with ARTI (age 12 days to ≤85 years) from four different locations in Sri Lanka: National Hospital, Kandy (NHK) (January 2021–October 2022); Teaching Hospital, Anuradhapura (THA) (March 2021–May 2021); National Cancer Institute Sri Lanka (NCISL) (January 2022–December 2024); and Teaching Hospital, Peradeniya (THP) (November 2023–December 2023). The study was approved by the Ethical Review Committee of the Faculty of Medicine, University of Peradeniya (2021/EC/21, 2022/EC/52), Post Graduate Institute of Science, University of Peradeniya (CEC-PGIS-2021–08) and the Medical Research Institute, Sri Lanka (ERC/ 2025/06). A total of 1,582 patients with ARTI symptoms, including fever (more than or equal to 38 °C) with, cough, cold, sore throat or shortness of breath within the first 7 days of the illness, were selected for the study among the samples received for routine laboratory testing. Demographic and clinical data were extracted from the patients’ clinical notes.

### Sample processing and RespiFinder 2Smart assay

The respiratory specimens were subjected to nucleic acid extraction using locally validated commercial kits (QIAGEN, Germany, or SpinStar, Malaysia, or Maxwell® RSC Viral Total Nucleic Acid Purification Kit and using the Maxwell® RSC48, USA). The nucleic acid extracts were tested for respiratory pathogens [(influenza-A, influenza-B, influenza virus H1N1 pdm 09, respiratory syncytial virus-A, respiratory syncytial virus-B, human parainfluenza virus-1, human parainfluenza virus-2 (hPIV-2), human parainfluenza virus-3 (hPIV-3), human parainfluenza virus-4, human coronavirus OC43 (hCoV OC43), human coronavirus 229E, human coronavirus NL63/HKU1, rhinovirus/enterovirus (Rh/EnV), human adenovirus (hAdV), hMPV, human bocavirus type-1 (hBoV-1) and four atypical bacteria such as *Mycoplasma pneumonia*, *Chlamydophila pneumoniae*, *Legionella pneumophila* and *Bordetella* species] by a commercial multiplex real-time PCR assay (RespiFinder2Smart, PathoFinder, catalogue no: PF2600-2S, Netherlands) according to the manufacturers’ guidelines.

### Illumina sequencing

A subset of hMPV-positive samples was then selected for genomic sequencing. Sequencing was performed using the advanced Illumina sequencing platform at the Genomics Laboratory, NCISL. A commercial respiratory virus sequencing kit (Illumina Respiratory ID/AMR Panel, Illumina, USA) was employed. The sequencing was performed according to the manufacturer’s instructions using the Illumina NextSeq 1000 System, known for its precision in high-throughput sequencing workflows. The forward and reverse sequences were assembled, and consensus contig assembly was performed using an Illumina BaseSpace sequence assembler V.2.0.0.

## Results

A total of 1,582 nasopharyngeal swab samples were tested. Of these, hMPV was detected in 26/1,582 (1.64%) patients. A summary of hMPV detection across different locations in Sri Lanka from 2021 to 2023 is shown in Table 1.

**Table 1.** hMPV infections detected from different areas of Sri Lanka from 2021 to 2024

Location	No. of samples tested (*n*=1582)	hMPV positive (*n*=26)	Study duration
NHK	1,021	20 (1.96%)	January 2021–October 2022
THA	384	0	March 2021–May 2021
NCISL	102	2 (1.96%)	January 2022–December 2024
THP	75	4 (5.33%)	November 2023–December 2023

**Fig. 1.**
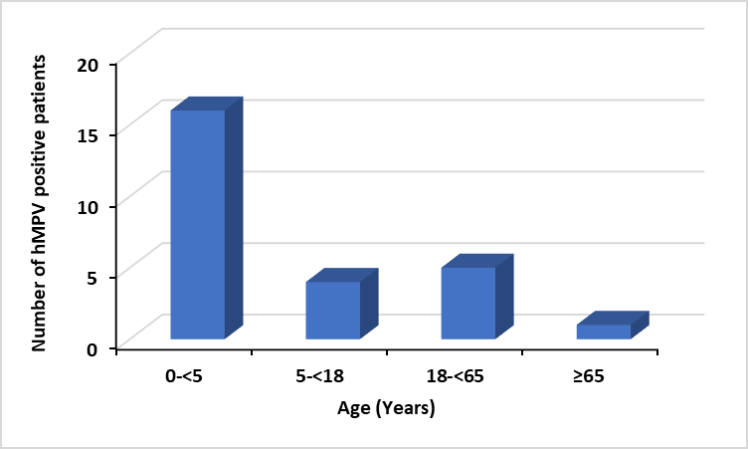
Distribution of hMPV infections across different age groups in the study sample.

hMPV infections were predominantly detected in children aged <5 years with a child-to-adult ratio of 10:3 among hMPV-positive patients. The infection was more prevalent in males (69.23%) than in females. Of the 26 hMPV-positive patients, 5 were co-infected with other respiratory viruses, including hBoV-1, hCoV OC43, hPIV-2 and Rh/EnV. All co-infected patients were under 5 years of age and two required admissions to the intensive care unit (ICU). The distribution of hMPV infection across different age groups is presented in Fig. 1.

The most common symptoms observed in hMPV-infected patients were fever, cough, cold and sore throat. Lower respiratory tract symptoms, including pneumonia and bronchiolitis, were noted in 12 (46.15%) patients, while 3 (11.53%) patients required ICU admission. All ICU-admitted patients were under 4 months of age and were immunocompromised. Clinical characterization of the hMPV-positive patients is summarized in Table 2.

**Table 2.** Clinical categorization of hMPV-positive patients in the present study sample

Clinical categorization		Total no. (%)	hMPV mono-infection (*n*=21)	hMPV co-infection (*n*=5)
Upper respiratory tract infection		14 (53.84)	12 (57.14)	2 (40)
LRTI	Bronchiolitis	2 (7.69)	1 (4.76)	1 (20)
	Bronchopneumonia	8 (30.77)	6 (23.07)	2 (40)
Severe infection with mortality*		2 (7.69)	2 (9.52)	0

*Both patients were adults and severely immunocompromised following chemotherapy.

Of the sequenced samples, only one was successfully sequenced and identified as hMPV type B on *L* gene analysis (GeneBank accession no. PV178132).

## Discussion

The present study findings provide details on the prevalence, demographic and clinical characteristics of hMPV infection in children and adults in different locations in Sri Lanka from 2021 to 2024. Overall prevalence of hMPV infection in this study was 1.64%, and this is lower than previously reported in Sri Lanka [14,16, 17]. A study done by Shapiro *et al.* among adults and children from March 2013 to January 2015 in the Southern part of Sri Lanka reports a 3.9% prevalence for hMPV infection [17]. Another study done by Jayaweera *et al.* in children less than 5 years in the North Central and Central provinces of Sri Lanka from March 2013 to August 2014 reported a prevalence of 3.3% in their study population [15]. Another study done by Noordeen *et al.* in 2019 identified hMPV as the most predominant virus responsible for SARI with a prevalence of 86% during a mini outbreak that occurred in 2019 [16]. It has also to be noted that the prevalence of hMPV in THP is higher than in the other locations in the current study, too. Moreover, the sample collection period of the THP was in the latter part of the Corona Virus Disease 2019 (COVID-19) pandemic. During the COVID-19 pandemic, non-pharmaceutical interventions (NPIs) like wearing face masks, regular hand washing, closure of schools and maintaining social distance would have contributed to the low prevalence in the other three locations, as the sampling in those was done during early or peak time of the pandemic prior to mid-2022. These NPIs were relaxed in mid-2022 in Sri Lanka following the COVID-19 vaccination. The relaxation of NPIs might have also influenced the increase in positivity rate in THP, apart from the higher positivity reported at the same location prior to the pandemic [18].

Low success rate for sequencing could be due to sub-optimal sample storage and transport from the peripheral laboratory to the sequencing laboratory during the COVID-19 pandemic, where laboratories were overwhelmed with a large number of samples.

Prevalence of hMPV infections was higher in males compared to females, and this finding is in agreement with other studies as well [10,19, 20]. Most of the hMPV-positive patients were children compared to adults, as has also been reported by Yi *et al.* in China [10]. However, the wider age distribution of hMPV is considerably varied from RSV infection which usually affects children <2 years of age and older adults >65 years of age [21]. These clinical symptoms ranged from mild upper respiratory tract infection to severe lower respiratory tract infections (LRTI), as discussed [10]. In our study, the most common symptoms observed were fever, cough and sore throat. Lower respiratory tract symptoms included pneumonia and bronchiolitis, and these were noted in 46.15% (*n*=12) of patients. Additionally, three patients (11.53%) required ICU care. These findings are in agreement with the findings of a case series reported by Jayaweera *et al.* in 2018 in Sri Lanka. In that study, hMPV infection in children has shown a range of respiratory symptoms with varying severity ranging from common cold to life-threatening LRTIs [14].

hMPV is often co-infected with other respiratory viruses, such as hAdV, RSV, RhV and hPIV [22,24]. In this study, the co-infection rate was 0.31%, with hPIV, which had the highest amount of co-infection with hMPV, as also noted by Fathima *et al.* in Alberta, Canada, in 2012 [25]. According to a case study conducted in Sri Lankan children with ARTI, the co-infection rate of hMPV-RSV infections has been high in Sri Lanka [14]. However, there were no hMPV-RSV co-infections noted in our study. Moreover, it has also to be noted that, in our study, 40% of co-infected patients required ICU care, and this is in agreement with previous studies, which concluded that the co-infection of hMPV with other respiratory viruses can aggravate clinical severity [26,27].

## Conclusion

In summary, hMPV infection was prevalent in 1.64% of the patients suspected of ARTI and the majority of the hMPV-infected patients were children less than 5 years of age. hMPV-infected patients showed a range of respiratory symptoms with varying severity, ranging from common cold to life-threatening LRTIs. hMPV co-infections have been noted with hBoV-1, hCoV OC43, hPIV-3 and Rh/EnV. Detailed and continuous screening for hMPV among adults and children will help to mitigate the global burden of hMPV, improve outcomes for high-risk populations and strengthen preparedness against respiratory viral infections leading to outbreaks, epidemics and pandemics.

## Supplementary material

10.1099/acmi.0.001022.v3 Uncited Table S1.

## Abbreviations

ARTIs: acute respiratory tract infections
COVID-19: Corona Virus Disease 2019
hAdV: human adenovirus
hBoV-1: human bocavirus type-1
hMPV: human metapneumovirus
hPIV-2: human parainfluenza virus-2
hPIV-3: human parainfluenza virus-3
ICU: intensive care unit
LRTI: lower respiratory tract infection
NCBI: National Center for Biotechnology Information
NCISL: National Cancer Institute Sri Lanka
NHK: National Hospital, Kandy
NPIs: non-pharmaceutical interventions
Rh/EnV: rhinovirus/enterovirus
SARI: severe acute respiratory tract infection
THA: Teaching Hospital, Anuradhapura
THP: Teaching Hospital, Peradeniya
