# Supplementary material for: A multi-centre study on epidemiology and clinical characteristics of human metapneumovirus infection in Sri Lanka from 2021 to 2024
Source: Access Microbiol. 2025 Aug 29;7(8):001022.v3. doi: 10.1099/acmi.0.001022.v3 (PMC12451297; doi:10.1099/acmi.0.001022.v3)
Supplement: Uncited Table S1. [file acmi-7-01022-s001.pdf]

Clinical and demographic details of hMPV positive patients in the study sample

Sample number	Hospital site	Age category (years)	Symptoms					Virus detected	Sample collection year-Month	Gender	Other pertinent information
			Fever	Cough	Sore throat	SOB	Diarrhea				
1	NHK	0-<5	Yes	Yes	No	No	No	hMPV	2021- Nov	M	Bronchopneumonia
2	NHK	5-<18	Yes	Yes	Yes	Yes	No	hMPV	2022- Jan	M	URTI
3	NHK	>=65	No	No	No	No	No	hMPV	2022 -Jan	F	URTI
4	NHK	0-<5	No	No	No	No	No	hMPV	2022 -Feb	M	URTI
5	NHK	0-<5	No	No	No	No	No	hMPV	2022 - Feb	M	URTI
6	NHK	0-<5	Yes	Yes	No	No	Yes	hMPV	2022 -Mar	M	Bronchopneumonia
7	NHK	5-<18	Yes	Yes	No	No	No	hMPV	2022- Mar	M	URTI
8	NHK	0-<5	Yes	Yes	No	Yes	No	hMPV	2022 -Apr	M	URTI
9	NHK	5-<18	Yes	Yes	No	No	No	hMPV	2022- Apr	M	URTI
10	NHK	18-<65	Yes	Yes	No	Yes	No	hMPV	2022 -May	F	Bronchopneumonia
11	NHK	0-<5	Yes	Yes	Yes	Yes	No	hMPV, hBoV-1	2022 -May	M	Bronchopneumonia, Immunocompromised
12	NHK	18-<65	No	No	No	No	No	hMPV	2022 - May	M	URTI
13	NHK	0-<5	No	No	No	No	No	hMPV	2022 -Jun	F	URTI
14	NHK	0-<5	No	No	No	No	Yes	hMPV, hBoV-1	2022 -Jun	M	URTI
15	NHK	18-<65	No	No	No	No	No	hMPV	2022 -Jun	M	URTI
16	NHK	0-<5	No	No	No	No	No	hMPV	2022 -Jun	M	URTI
17	NHK	0-<5	No	No	No	No	No	hPIV2, hMPV	2022 -Jun	M	URTI
18	NHK	0-<5	No	No	No	No	No	hMPV	2022- Oct	F	URTI
19	NHK	5-<18	Yes	Yes	Yes	No	No	hMPV	2022- Oct	F	Bronchopneumonia
20	NHK	0-<5	Yes	Yes	No	Yes	No	hMPV, hCoV-OC43	2022 -Oct	M	Bronchopneumonia
21	NCI	18-<65	Yes	Yes	No	No	No	hMPV	2022-2024	M	Pneumonia, Immunocompromised
22	NCI	18-<65	Yes	Yes	No	No	No	hMPV	2022-2024	F	Pneumonia, Immunocompromised
23	THP	0-<5	Yes	Yes	No	No	No	hMPV, Rh/En	2023	F	Moderate bronchiolitis
24	THP	0-<5	Yes	Yes	No	No	No	hMPV	2023	M	Bronchopneumonia
25	THP	0-<5	Yes	Yes	No	No	No	hMPV	2023	M	Moderate bronchiolitis
26	THP	0-<5	Yes	Yes	No	No	No	hMPV	2023	F	Bronchopneumonia

NHK-National Hospital Kandy; NCI- National Cancer Institute; THP-Teaching Hospital Peradeniya; Y-Year; M-Month; M-Male; F- Female; SOB-Shortness of Breath; URTI-Upper Respiratory Tract Infection
